# A Reconfigurable, Dual-Output INHIBIT and IMPLICATION Molecular Logic Gate

**DOI:** 10.3389/fchem.2020.00470

**Published:** 2020-06-05

**Authors:** Lavinia A. Trifoi, Gregory K. Hodgson, Nicholas P. Dogantzis, Stefania Impellizzeri

**Affiliations:** Laboratory for Nanomaterials and Molecular Plasmonics, Department of Chemistry and Biology, Ryerson University, Toronto, ON, Canada

**Keywords:** fluorescence, molecular switches, photochemistry, molecular logic gates, boron dipyrromethene, protonation

## Abstract

Molecules that respond to input stimulations to produce detectable outputs can be exploited to mimic Boolean logic operators and reproduce basic arithmetic functions. We have designed a two-state fluorescent probe with tunable emission wavelength for the construction of a molecular logic gate with reconfigurable single– or dual–output capability. The system is based on a BODIPY skeleton coupled with 4-(dimethylamino)benzaldehyde. The behavior of the molecular logic gate can be easily investigated in solution with fluorescence spectroscopy, and the optical readout (fluorescence) can be monitored in one (green) or two (green and red) channels. Depending on the solvent of choice, single INHIBIT or dual INHIBIT/IMPLY logic functions can be achieved using chemical inputs (acid and base). Reconfiguration from single– to dual–output is thus made possible by operating the system in acetonitrile (single output) or toluene (dual output), respectively. The logic gate can be switched by manipulating the fluorescence emission via protonation or deprotonation, even when immobilized onto a glass substrate. At the solid state, the resulting output can be stored for extended periods of time. This feature provides two added benefits: (i) memory function and (ii) “set/reset” capability of the logic gate. Our design thus provides a proof-of-concept interface between the molecular and electronic domains.

**Graphical Abstract:**
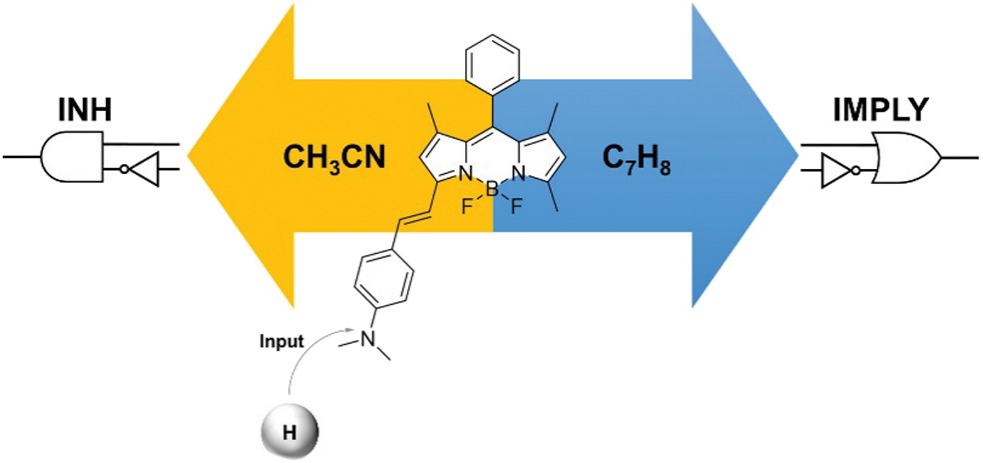


## Introduction

The miniaturization race in the computer industry continues to deliver faster processors, larger capacity memory and electronic circuits with higher chip densities and computational power (International Roadmap for Devices and Systems Executive Summary, 2018). The “top-down” fabrication of smaller electronic devices and components is, however, rapidly approaching a limit due to the fact that the properties of bulk semiconductors vanish at nanometer dimensions (Vollath, 2013; Shi, 2015; Zhang, 2018; Ghatak and Mitra, 2019). Nevertheless, relentless global demand for faster data transmission and storage capacity underpins the need to investigate new materials for encoding large volumes of data at the nanoscale (Sarid and Schechtman, 2007; Reinsel et al., 2018). In computers, information processing is based on Boolean (binary) algebra. In this two-state logic framework, variables can assume only one of two values (1 or 0) at a given time. Digital electronics require the encoding of data as electrical and optical signals in the form of such binary digits, and rely on the utilization of interconnected logic gates, i.e., idealized or physical devices implementing one or more Boolean functions (Hwang, 2016; Deschamps et al., 2017). A logic gate executes a logical operation on one or more binary and produces a single binary output according to convention (Gibson, 2013). Driven by the demand for higher performance capabilities, computer technology continues to stimulate the exploration of innovative materials and methodologies for the construction of logic gates. To this end, extension of information processing and computation to the molecular level can be achieved through the development of a molecular-level electronic set capable of executing functions that mimic those performed by macroscopic components (Sun et al., 2014; Mathew and Fang, 2018).

Organic molecular switches can adjust their structural, electronic and spectroscopic properties when stimulated with chemical or photonic inputs and, in return, produce a chemical, electrical and/or optical output that varies with the switching process (Feringa and Browne, 2011). The resemblance between molecular switches and logic gates is clear: they can both convert input stimulation into output signals. It follows that the principles of Boolean algebra can be applied to signal transduction operated by molecular switches. Indeed, molecular switches have long been considered valuable candidates for the realization of user-programmable electronic devices, and examples have been reported for basic (AND, NOT, and OR) and more complex (NOR, XOR, XNOR) logic functions (de Silva and Uchiyama, 2007; de Silva, 2012a,b; Szaciłowski, 2012; Stojanovic et al., 2014; Andréasson and Pischel, 2015; Erbas-Cakmak et al., 2018). In this context, switchable fluorescent dyes that are sensitive to external chemical stimuli provide convenient platforms for the fabrication of sophisticated molecular devices that communicate through changes in their emission properties (e.g., wavelength, quantum yield) (Daly et al., 2014). In addition, changes in emission spectra can be simultaneously observed at more than one wavelength (dual–output), resulting in the possibility of implementing more than one logic function in a single, unimolecular system (Baytekin and Akkaya, 2000; de Silva and McClenaghan, 2002; Margulies et al., 2004; Coskun et al., 2005; Shiraishi et al., 2005; Jiang and Ng, 2014; Swaminathan and Balasurbamanian, 2019).

**Scheme 1:**
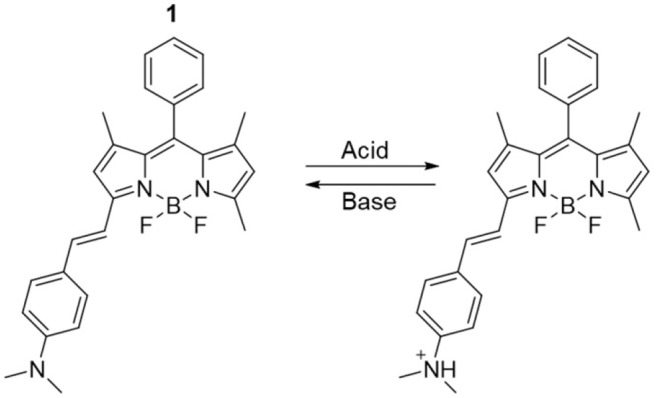
The two-state fluorescent molecular switch.

Upon the basis of these considerations, we targeted the 3-(dimethylamino)styryl-substituted boron dipyrromethene (BODIPY) dye **1** (Scheme 1). Due to their easily accessible structural modifications and remarkable photophysical properties such as photostability, high quantum yields and absorption coefficients, BODIPY chromophores have been successfully used for the development of selective and efficient chemosensors (Jeong and Yoon, 2012; Leen and Dehaen, 2012; Costero et al., 2018; Kaur and Singh, 2019). Extension of the BODIPY core conjugation by condensation with dialkylaminobenzaldehydes, as in **1**, shifts the emission to higher wavelengths and allows the installation of a pH-sensitive appendix that produces distinguishable changes in fluorescence upon protonation/deprotonation (Rurack et al., 2001; Baruah et al., 2006; Yu et al., 2006; Kulyk et al., 2017). Such switchable behavior has been previously exploited to construct an INHIBIT (INH) operator at the molecular level using a phenolic analog of **1** in solution (Coskun et al., 2005). Herein, we considerably expanded upon the logic capabilities of **1** by taking advantage of the solvent-dependency of its quantum yield to simultaneously achieve INH and IMPLY (logical implication) processing. The behavior of the molecular logic gate can be easily investigated in solution with fluorescence spectroscopy. Moreover, we imparted a memory function into the system by immobilizing it on a solid glass substrate, where the resulting output could be stored for extended periods of time.

## Materials and Methods

### Synthesis

Syntheses were adapted from previously published protocols (Rurack et al., 2001; Coskun et al., 2005). All reaction schemes are illustrated in the Electronic Supplementary Material.

#### Synthesis of 4,4-Difluoro-1,3,5,7-tetramethyl-8-phenyl-4-bora-3a,4a-diaza-s-indacene (0)

In a 1000 mL round bottom flask, 2,4-dimethylpyrrole (500 mg, 5.2 mmol), benzaldehyde (276 mg, 2.6 mmol) and trifluoroacetic acid (TFA, 3 drops), were dissolved in 300 mL of CH_2_Cl_2_ and placed under N_2_ atmosphere. The solution was degassed for 20 min, then stirred under nitrogen at room temperature overnight to ensure complete consumption of benzaldehyde. During the process of degassing, a color change from yellow to burnt orange to red was observed. Once TLC confirmed that benzaldehyde was consumed, a CH_2_Cl_2_ solution (100 mL) of DDQ (590 mg, 2.6 mmol) was added to the reaction mixture under air. After stirring for 20 min, 3 mL of NEt_3_ were added followed by 3 mL of BF_3_ OEt_2_. After an additional 30 min of stirring, the reaction was extracted three times with 1:1 H_2_O:CH_2_Cl_2_ and once with 1:1 brine:CH_2_Cl_2_. The combined organic phases were dried over Na_2_SO_4_ and evaporated under reduced pressure, resulting in a dark green solid. The crude compound was purified by column chromatography [SiO_2_:Hexanes/EtOAc 7:1 (v/v) + 1% NEt_3_], affording a bright orange solid in 45.8% yield. ^1^H NMR (400 MHz, CDCl_3_) δ 7.50 (d, 3H), 7.30 (d, 2H), 6.00 (s, 2H), 2.58 (s, 6H), 1.40 (s, 6H); EI-MS calculated for C_19_H_19_BF_2_N_2_ (M^+^) 324.16; found 324.1.

#### Synthesis of 1

To a clean 500 mL round bottom flask, compound **0** (735 mg, 0.33 mmol), 4-(dimethylamino)benzaldehyde (49.2 mg, 0.33 mmol), toluene (137 mL), glacial acetic acid (1.71 mL) and piperidine (2.05 mL) were added. The solution was refluxed for 72 h, using a Dean-Stark apparatus to remove any water formed. The crude compound was concentrated under reduced pressure and purified by column chromatography [SiO_2_:Hexanes/EtOAc 7:1 (v/v)]. The blue fraction (9.4% yield) was collected and used for spectroscopic investigations. ^1^H NMR (400 MHz, CDCl_3_) δ 7.51–7.49 (m, 6H), 7.32–7.30 (t, 2H), 7.23–7.19 (d, 2H), 6.67–6.72 (m, 2H), 6.59 (s, 1H), 5.96 (s, 1H), 3.03–3.04 (d, 6H), 2.59 (s, 3H), 1.42 (s, 3H), 1.38 (s, 3H).

#### Synthesis of 4,4-Difluoro-8(4'-hydroxyphenyl)-1,3,5,7-tetramethyl-4-bora-3a,4a-diaza-s-inda-cene (0b)

To a clean 1000 mL round bottom flask, 2,4-dimethylpyrrole (500 mg, 5.26 mmol), 4-hydroxybenzaldehyde (320 mg, 2.6 mmol), TFA (3 drops) and CH_2_Cl_2_ (300 mL) were added. The reaction was placed under N_2_ atmosphere and degassed for 20 min. A color change from yellow to orange to deep red was observed. The solution was stirred at room temperature under inert conditions for 24 h. Once the consumption of 4-hydroxybenzaldehyde was confirmed by TLC, DDQ (590 mg, 2.6 mmol) dissolved in 100 mL of CH_2_Cl_2_ was added to the reaction mixture under air. The solution was stirred for 30 min followed by addition of NEt_3_ (3 mL) and then BF_3_ OEt_2_ (3 mL). After 30 min of stirring, the solution was washed three times with 1:1 H_2_O:CH_2_Cl_2_ and once with 1:1 brine:CH_2_Cl_2_. The combined organic layers were dried over Na_2_SO_4_, evaporated under reduced pressure and used for the next step without further purification. Yield 38.5%. ^1^H NMR (400 MHz, CDCl_3_) δ 7.53 (d, 2H), 6.95 (d, 2H), 6.59 (s, 3H), 3.02-3.09 (t, 6H), 2.48 (s, 3H), 1.45 (s, 3H), 1.42 (d, 2H).

#### Synthesis of 2

In a clean, dry 1000 mL flask, compound **0b** (310 mg, 0.91 mmol) and 4-(dimethylamino)benzaldehyde (136 mg, 0.91 mmol) were dissolved in toluene (65 mL), followed by the addition of glacial acetic acid (0.7 mL), and piperidine (0.9 mL). The solution was refluxed for 72 h, using a Dean-Stark apparatus to remove any water formed during the reaction. The crude compound was concentrated under reduced pressure and purified by column chromatography [SiO_2_: Hexanes/EtOAc 1:1 (v/v) then CH_2_Cl_2_/MeOH 95:1 (v/v)] The blue fraction was collected and used immediately for the next step (4.2% yield). FTIR-ATR: 3330 cm^−1^ (ν_s_, O-H); 3000, 2930, 2885 cm^−1^ (ν_s_, C–H); 1636 cm^−1^ (ν_s_, C=C, alkene and aromatic); 1560 cm^−1^ (ν_b_, N–H); 1447 cm^−1^ (ν_s_, aromatic C=C); 1390 cm^−1^ (ν_b_, CH_3_); 1265 cm^−1^ (ν_s_, C–N aromatic); 1060 cm^−1^ (ν_s_, C–O); 947 cm^−1^ (ν_b_, O–H); 766 cm^−1^ (ν_b_, C–H, oop).

#### Synthesis of 3

In a clean 100 mL two-neck round bottom flask, a stir bar and compound **2** (3.5 mg, 7.4 μmol) were added. The system was placed under N_2_ atmosphere and allowed to degas for 30 min followed by the addition of dry THF (18.7 mL). Maintaining inert conditions, 3-(triethoxysilyl)propyl isocyanate (37.4 μL, 151 μmol) was added and the reaction was refluxed at 70°C for 24 h. The reaction mixture was then concentrated under reduced pressure (mass obtained: 13.7 mg) and used for functionalization of glass slides without further purification. FTIR-ATR: 3000, 2910, 2870 cm^−1^ (ν_s_, C–H); 1720 cm^−1^ (ν_s_, C=O); 1600 cm^−1^ (ν_s_, C=C, alkene and aromatic); 1515 cm^−1^ (ν_b_, N–H); 1456 cm^−1^ (ν_s_, aromatic C=C); 1380 cm^−1^ (ν_b_, CH_3_); 1264 cm^−1^ (ν_s_, C–N aromatic); 1113 cm^−1^ (ν_s_, Si–CH_2_); 1065 cm^−1^ (ν_s_, Si–OEt); 1040 cm^−1^ (ν_s_, C–O); 806, 740 cm^−1^ (ν_b_, C–H, oop).

### Methods

2,4-dimethylpyrrole, benzaldehyde, 2,3-dichloro-5,6-dicyano-1,4-benzoquinone (DDQ) and boron trifluoride diethyl etherate (BF_3_ OEt_2_) were purchased from Sigma Aldrich. Solvents and trifluoroacetic acid (TFA) were purchased from ACP. Dry CH_2_Cl_2_ and THF were purified with an MBraun MBM-SPS solvent purification system. Ultrapure deionized water (MilliΩ, 18.2 MΩ) was obtained from a Millipore Purification System. Reactions were monitored by thin layer chromatography using aluminum backed sheets coated with 200 μm silica (60, F_254_). SiliaFlash® P60, 40–63 mm (230–400 mesh) silica gel from SiliCycle was used for purification of compounds by column chromatography. Mass spectral analysis was performed with a 7890B GC System equipped with a 5977 mass selective detector from Agilent Technologies. NMR spectra were recorded at room temperature with a Bruker Avance 400 spectrometer. Steady-state absorption spectra were recorded with an Agilent Cary 60 UV-visible spectrometer, using quartz cells with a path length of 1 cm. Steady-state emission spectra of aerated solutions as well as functionalized and unfunctionalized glass slides were recorded with an Agilent Cary Eclipse spectrometer operating in right-angle geometry. Diffuse reflectance measurements were performed using an Agilent Cary 5000 spectrophotometer equipped with a DRA-2500 accessory. FTIR Diamond ATR spectra were recorded with a Cary 630 spectrometer by Agilent Technologies. Glass slides were soaked in piranha solution (3:1 H_2_SO_4_:H_2_O_2_) for 1 h followed by several rinses with deionized and MilliΩ water, respectively. The slides were then stored in MilliΩ water for future use. Crude compound **3** (13.7 mg) was then dissolved in 1 mL of toluene and 300 μL of this solution was drop casted onto each slide. Wet slides were allowed to dry inside a fume hood for 5 days until all the solvent evaporated. Functionalized slides were thoroughly rinsed with toluene and subjected to one full acid/base cycle for equilibration.

## Results and Discussion

### Solution Spectroscopy (Combinatorial Logic)

**Figure 1:**
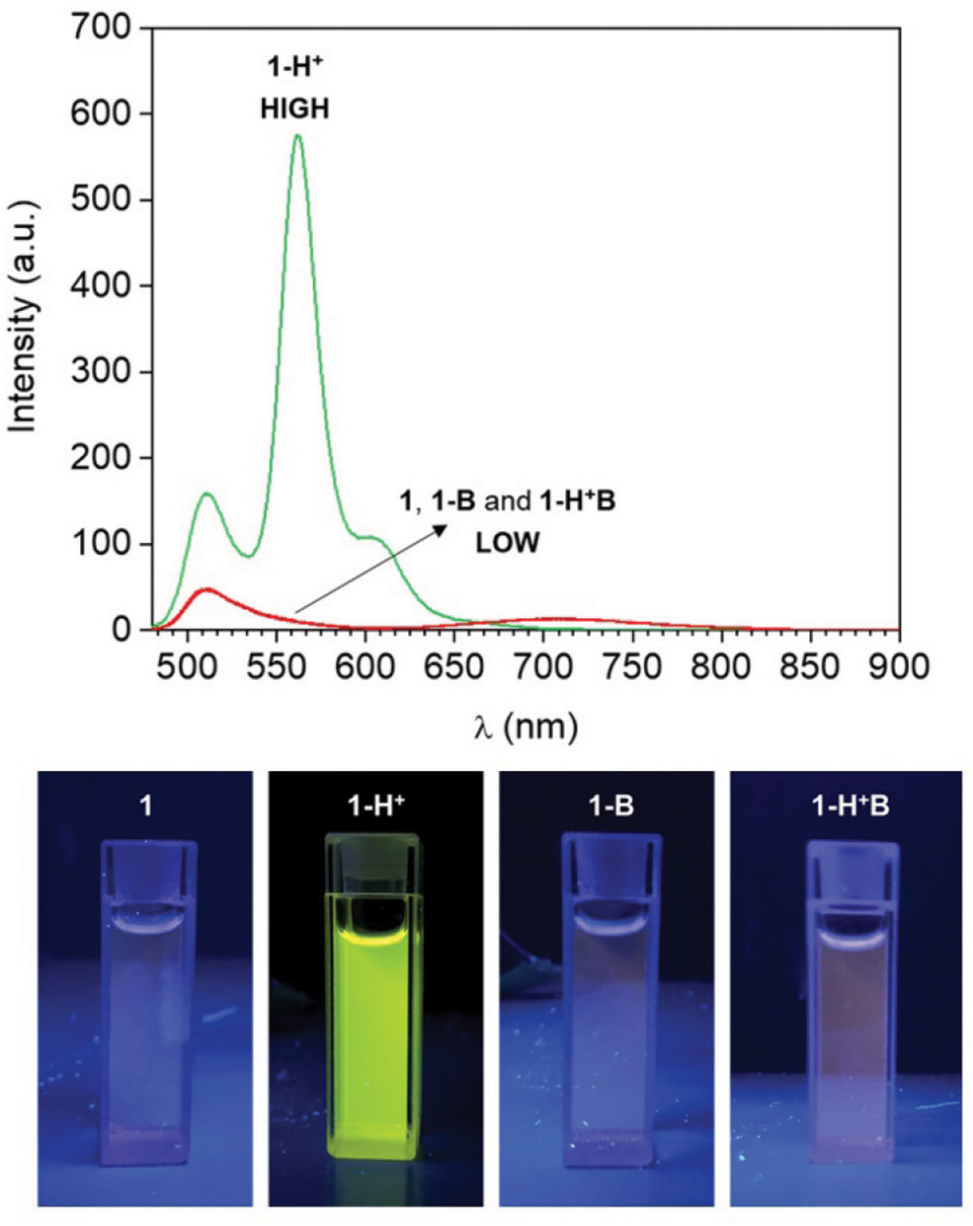
**(Top)** Emission spectra of a CH_3_CN solution of **1** (1 μM, 20°C, λ_Ex_ = 460 nm) before (**1**) and after the addition of 50 eq. HClO_4_ (**1-H**^**+**^), 50 eq. NEt_3_ (**1-B**) and 50 eq. of HClO_4_ + 50 eq. NEt_3_ (**1-H**^**+**^**B**). **(Bottom)** Digital photographs of the same solutions illuminated at 365 nm with a laboratory UV lamp.

Compound **1** is based on a BODIPY fluorophore, where a 4-(dimethylamino)styryl group was used to replace one of the pyrrolic methyls. This transformation extends the conjugation of the adjacent BODIPY core, shifting its absorption band bathochromically to λ_Abs_ = 598 nm (Figure S1, Electronic Supplementary Material). Spectroscopic data from our (Figure 1) and other (Rurack et al., 2001; Baruah et al., 2006; Yu et al., 2006) laboratories have shown that a broad emission band for **1** in CH_3_CN appears at λ_Em_ = 710 nm due to an intramolecular charge transfer (ICT) process between the tertiary amino group, which possesses strong electron donating properties, and the electron-deficient boron-dipyrromethene. In addition, the fluorescence of **1** is almost completely quenched in CH_3_CN (***1***in Figure 1) due to accelerated internal conversion (Baruah et al., 2006). Protonation of the tertiary amine upon addition of 50 equivalents of HClO_4_ to **1** dramatically alters its electron donating properties and consequently suppresses the charge transfer interaction. As a result, both absorption (Figure S1) and emission (Figure 1) spectra of **1-H**^**+**^ shift toward shorter wavelengths (λ_Abs_ = 550 nm and λ_Em_ = 565 nm), mimicking the typical BODIPY-like narrow, structured bands. In contrast to its unprotonated form, **1-H**^**+**^ is highly fluorescent. Reversible deprotonation of **1-H**^**+**^ to **1** upon the addition of 50 equivalents of triethylamine (NEt_3_) restores the original bands (***1-H***^**+**^***B***in Figure 1, Figure S1), while prior addition of NEt_3_ to the unprotonated dye (***1-B***in Figure 1 and Figure S1) has no effect on its spectroscopic properties. This behavior is also illustrated by digital photographs of the same solutions illuminated at 365 nm with a laboratory UV lamp (Figure 1).

**Figure 2:**
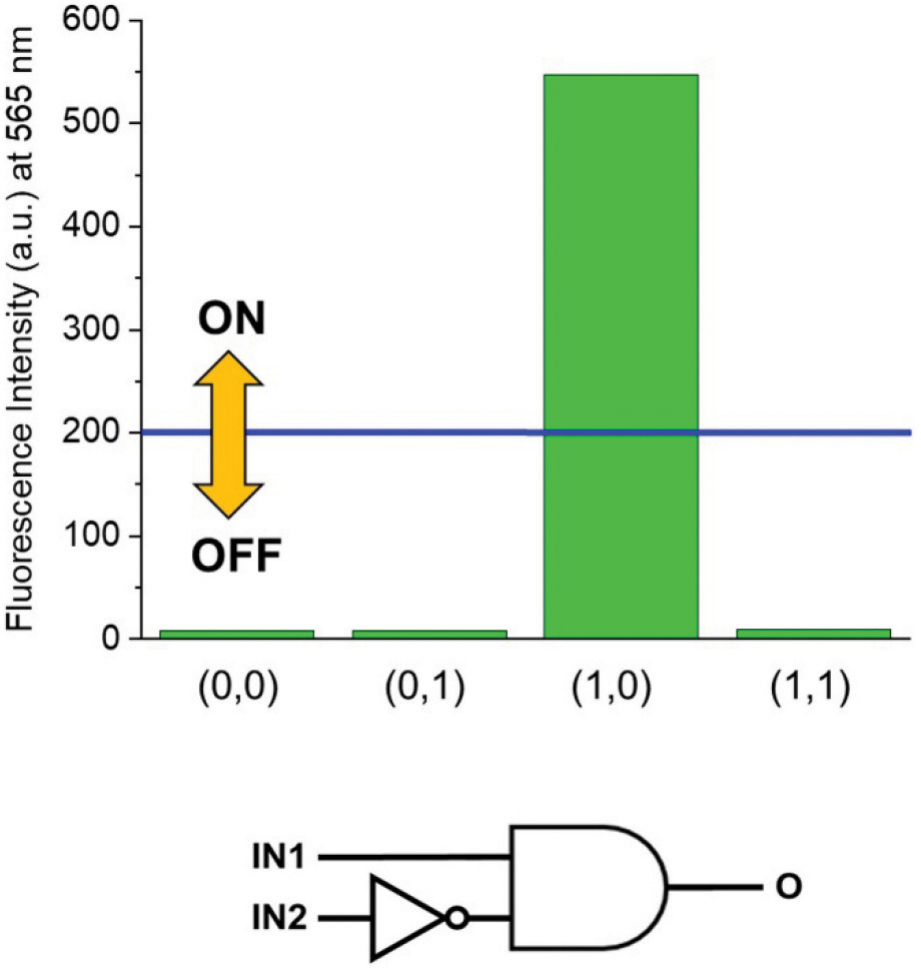
**(Top)** Column diagram of the fluorescence intensities at 565 nm. The blue line shows the ON-OFF threshold. **(Bottom)** The INH logic circuit for the two-state molecular switch when operated in CH_3_CN.

HClO_4_ (Input 1, IN1) and NEt_3_ (Input 2, IN2) can be either absent (OFF) or present (ON). Binary digits can thus be encoded such that the acid and base inputs can be logically processed using positive logic convention (OFF = 0 and ON = 1). Under our excitation conditions, the fluorescence intensity of **1** at 565 nm (Output, O) was measured at approximately 10 a.u. in the unprotonated form, but grows to 550 a.u. in the presence of acid (Figure 1). Assigning a threshold of 200 a.u. (*I* < 200 a.u. = LOW and *I* ≥ 200 a.u. = HIGH) and once again applying positive logic conventions (LOW = 0, HIGH = 1), this chemical system responds to an input string of two binary digits (IN1 and IN2) producing a binary output (O). For example, the input string (*0, 0)* indicates that both input stimuli, acid and base, are absent. Under these conditions, **1** is in its unprotonated form and the emission in the green channel (at 565 nm) is below the ON-OFF threshold; the output is 0. In contrast, an input string (*1, 0)* indicates that the acid input is present. Under these conditions, the dominant species is **1-H**^**+**^ and the fluorescence at 565 nm is high; the output is 1. Following this convention, the four output strings corresponding to the four possible combinations of input strings can be determined (Table 1 and Figure 2). From Table 1, it can be observed that the logic behavior of the two-state molecular switch in CH_3_CN corresponds to that of a combinatorial INH logic circuit (Figure 2).

**Table 1:** Truth table of the two-state molecular switch in CH_3_CN^*a*^−^*c*^.

**Input data**		**Output data**
**IN1**	**IN2**	**O**
0	0	0
0	1	0
1	0	1
1	1	0

a *The switching process is illustrated in Scheme 1*.

*^b^ The two inputs IN1 and IN2 are acid (H^+^) and base (B). The input strings where IN1 and IN2 are both 1 imply that acid and base are both present*.

c *The output O is the fluorescence at 565 nm. A positive logic convention (off = 0, on = 1) was applied to encode the binary digits for the two inputs and the output*.

**Figure 3:**
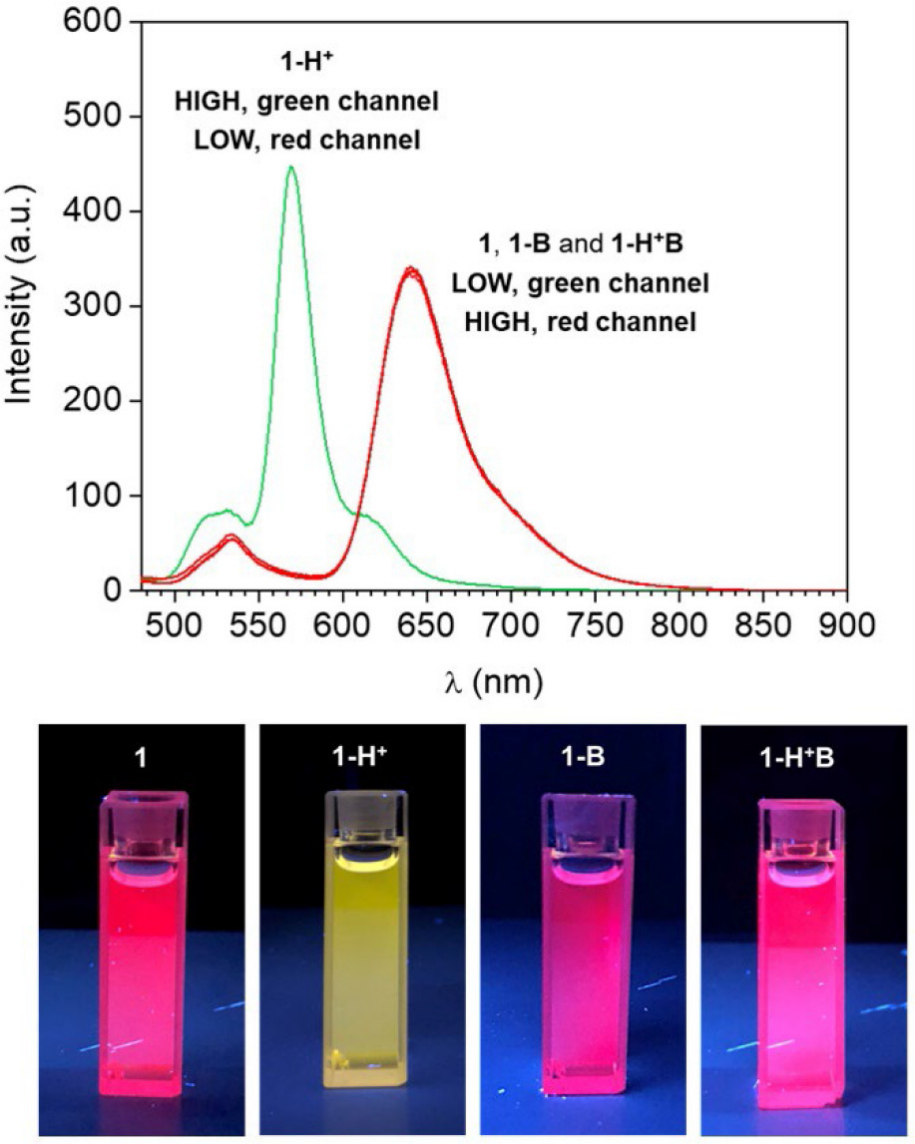
**(Top)** Emission spectra of a C_7_H_8_ solution of **1** (1 μM, 20°C, λ_Ex_ = 460 nm) before (**1**) and after the addition of 50 eq. HClO_4_ (**1-H**^**+**^), 50 eq. NEt_3_ (**1-B**) and 50 eq. of HClO_4_ + 50 eq. NEt_3_ (**1-H**^**+**^**B**). **(Bottom)** Digital photographs of the same solutions illuminated at 365 nm with a laboratory UV lamp.

Interestingly, when the molecular switch **1** is operated in CH_3_CN, the fluorescence in the red wavelength channel is excluded by the molecular processor, due to the low quantum yield of **1** in its unprotonated form (**1**, **1-B** or **1-H**^**+**^**B**, Figure 1). Nevertheless, the nonradiative quenching constant *k*_nr_ reduces from 7.58 × 10^8^ s^−1^ in CH_3_CN to 0.46 × 10^8^ s^−1^ in C_7_H_8_ (Baruah et al., 2006). In agreement with these data, as well as other examples of polarity-sensitive dyes for which emission is substantially increased with a decrease in solvent polarity (Wagner et al., 2018), the emission spectrum of compound **1** dissolved in toluene (***1***in Figure 3) displays a strong fluorescence band. Noticeably, the emission maximum shifts hypsochromically from 710 nm in CH_3_CN (polar solvent) to 640 nm in C_7_H_8_ (non-polar solvent) and is accompanied by a gain of fine structure and a decrease in bandwidth. This is a well-known phenomenon for (dimethylamino)styryl boron dipyrromethene dyes. Quantum mechanical calculations (Baruah et al., 2006) have confirmed that an ICT process is responsible for the observed solvent sensitivity, with polar aprotic solvents such as CH_3_CN stabilizing the excited state and thus red-shifting the emission. Similarly to CH_3_CN, subjecting **1** to protonation by addition of 50 equivalents of HClO_4_ in C_7_H_8_ switches the absorption (Figure S2) and emission (***1-H***^**+**^ in Figure 3) to lower wavelengths, as evidenced by the appearance of a strong fluorescence band at 565 nm and almost complete disappearance of the red fluorescence at 640 nm. Similar to the behavior observed in acetonitrile, addition of NEt_3_ to the unprotonated dye (***1-B***in Figure 3, Figure S2) has no remarkable effect on its spectroscopic properties, while deprotonation of **1-H**^**+**^ to **1** reinstates the original bands (***1-H***^**+**^***B*** in Figure 3, Figure S2).

**Figure 4:**
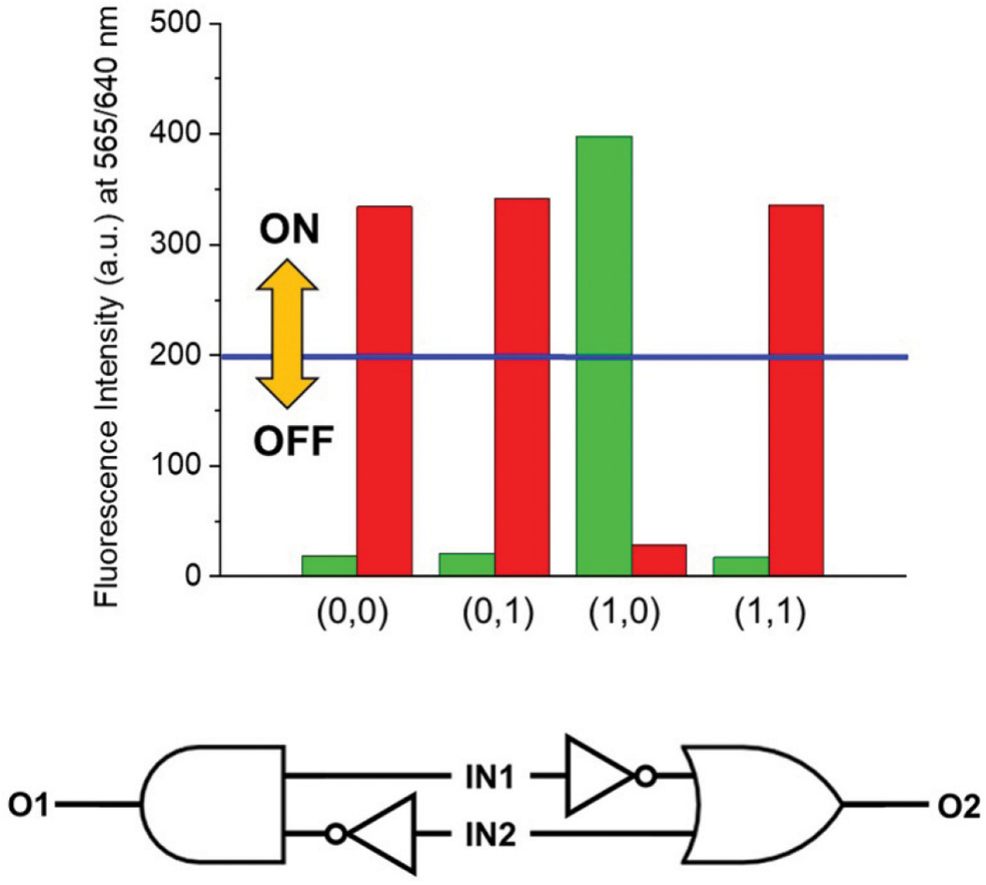
**(Top)** Column diagram of the fluorescence intensities at 565 (green columns) and 640 (red columns) nm. The blue line indicates the ON-OFF threshold. **(Bottom)** The dual–output combinatorial logic circuit INH/IMPLY representative of the two-state molecular switch when operated in C_7_H_8_.

As visibly shown by the digital photographs reported in Figure 3, the dual fluorescence of **1** in C_7_H_8_ translates into the possibility of simultaneously monitoring two separate outputs. Indeed, a change in emission above the set threshold can now be recorded at two different channels, λ_Em_ = 565 nm (Output 1) or λ_Em_ = 640 nm (Output 2). Using the same threshold value of 200 a.u. (*I* < 200 a.u. = LOW and *I* ≥ 200 a.u. = HIGH) and applying positive logic convention (LOW = 0, HIGH = 1), this chemical system responds to an input string of two binary digits (IN1 and IN2) producing two binary outputs (O1 and O2) in toluene. The four output strings corresponding to the four possible combinations of input strings can be determined for the two outputs independently (Table 2). The results presented in Table 2 illustrate that the logic behavior of the two-state molecular switch in C_7_H_8_ corresponds to that of a combinatorial INH logic circuit when the output is monitored in the green channel (O1), or as an IMPLY (Figure 4) circuit when tracked in the red channel (O2). The molecular operator can thus be instantaneously reconfigured simply by the selection of monitoring wavelength.

**Table 2:** Truth table of the two-state molecular switch in C_7_H_8_^*a*^−^*c*^.

**Input data**		**Output data**	
**IN1**	**IN2**	**O1**	**O2**
0	0	0	1
0	1	0	1
1	0	1	0
1	1	0	1

a *The switching process is illustrated in Scheme 1*.

*^b^ The two inputs IN1 and IN2 are acid (H^+^) and base (B). The input strings where IN1 and IN2 are both 1 imply that acid and base are both present*.

c *>Output O1 is fluorescence at 565 nm, while output O2 is fluorescence at 640 nm. A positive logic convention (off = 0, on = 1) was applied to encode the binary digits for the inputs and outputs*.

### Solid State Spectroscopy (Sequential Logic)

**Figure 5:**
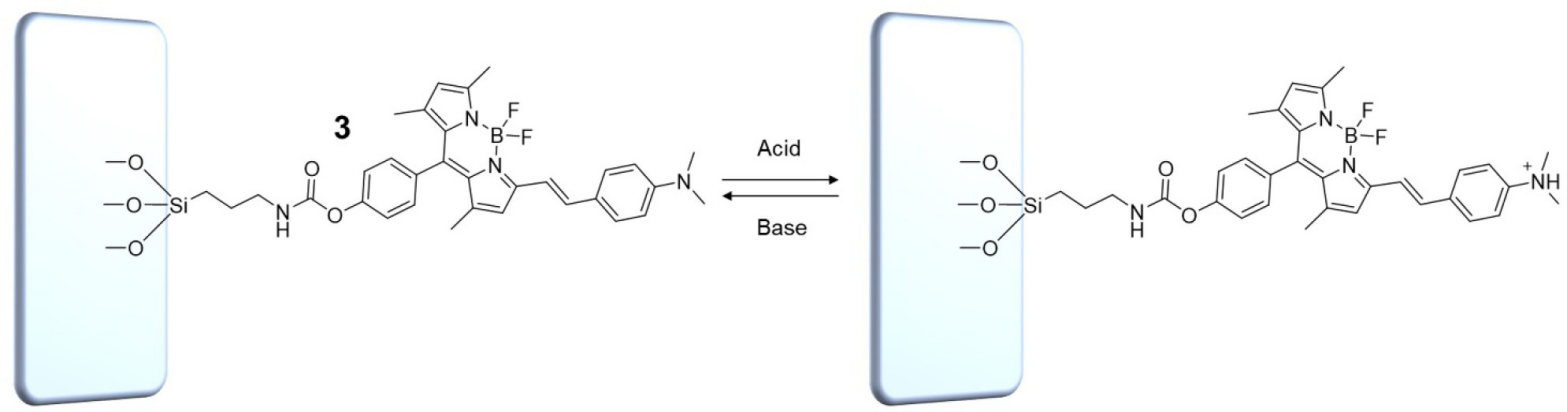
Protonated and unprotonated forms of the molecular switch **3** immobilized on a solid glass support.

In digital circuit theory, *combinational logic* refers to circuits whose outputs are a function of the present value of the inputs only (Lipiansky, 2012). In other words, the outputs obtained by the logic operator are purely a function of the inputs. If the inputs are changed, the outputs are changed (time independent logic). It follows that in combinatorial systems, any information about the previous status of the inputs is lost. Combinational logic circuits have no memory function because the current output state cannot be stored, and thus has no consequence for the next resulting output state. No feedback is required; the operator's output simply reflects the current status of the inputs according to the logic convention obeyed by the logic gate. Consequently, such systems cannot be used to store data. Upon the basis of these considerations, the behavior of the molecular switch **1** in solution can be classified as combinatorial logic. Even though the choice of solvent system (CH_3_CN vs. C_7_H_8_) determines the particular arithmetic capabilities of the logic gate, its behavior lacks memory regardless. In contrast, the output of sequential logic circuits depends not only upon the latest inputs, but also on the condition of earlier inputs. In *sequential logic*, the output is a function of the present inputs as well as the previous states of the system, and involves feedback from output to input that is stored in the system's memory for the next operation (Lipiansky, 2012). Such systems can be used to store data for extended periods of time. To implement sequential character into the molecular logic gate **1**, its chemical structure was synthetically modified by insertion of a hydroxyl group (**2**, ESI) followed by reaction with 3-(triethoxysilyl)propyl isocyanate (ICPTES) to yield compound **3**. Successful modification of **2** with the silane cap was confirmed by FTIR-ATR spectroscopy (Figure S3). Specifically, the FTIR-ATR spectrum of **3** shows the appearance of a peak at ~1720 cm^−1^ attributable to the C=O stretching vibration and the sharp, well-defined doublet for the stretching absorptions of Si–CH_2_ at 1065 and Si–OEt at 1110 cm^−1^. Furthermore, the OH stretching absorption at 3330 cm^−1^, clearly identified in the spectrum of **2**, disappears in that of the capped molecule. Without further purification, compound **3** was dissolved in C_7_H_8_ and drop casted onto a clean glass slide (Figure 5). The slides were subsequently washed with C_7_H_8_ to remove any unbound **3** or trace amounts of residual **2**, then dried prior to use. The presence of **3** was confirmed by diffuse reflectance spectroscopy (Figure S4). Note that Figure 5 illustrates the mode of chemical attachment of **3** to the glass support, but should not be interpreted as conclusive evidence of the formation of a uniform or non-uniform monolayer. Nevertheless, the number of dye molecules immobilized on the glass support was estimated to be on the order of 10^13^ cm^−2^, which is within the expected range for a non-uniform monolayer (ESI).

**Figure 6:**
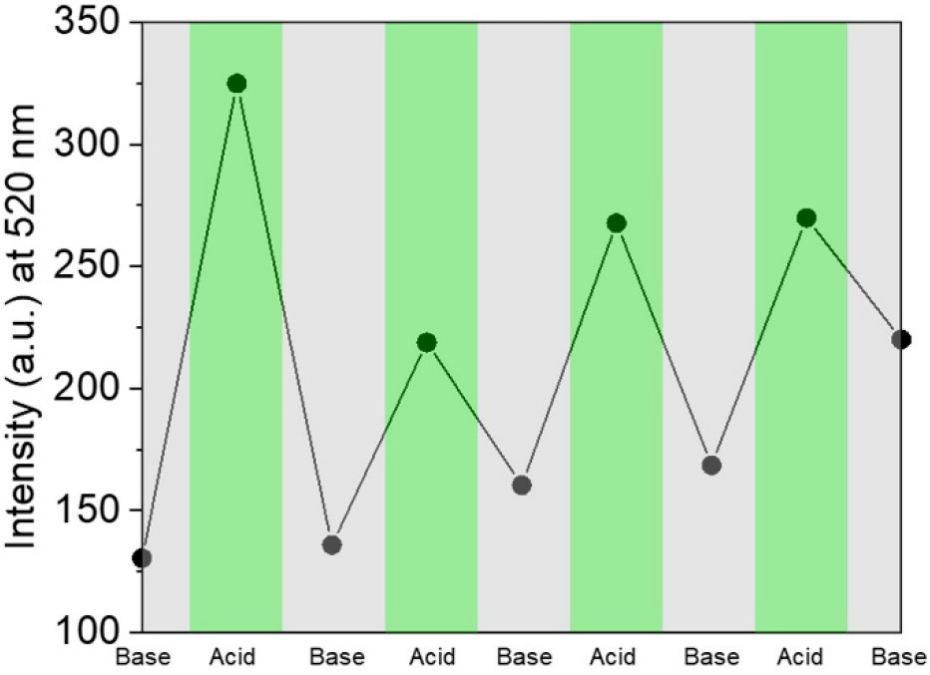
Fluorescence intensity recorded at 520 nm upon several protonation/deprotonation cycles performed upon **3** immobilized on a glass slide.

With our design, protonation/deprotonation of the molecular switch allows for effective fluorescence modulation at the solid state. Specifically, the fluorescence of **3** is partially quenched (***3***in Figure S5) as a result of the formation of aggregated clusters, which favors non-radiative decay processes from the excited state due to π-π stacking. After functionalized glass slides were immersed in 15 mM HClO_4_ in ethanol for 30 min, and subsequently washed with EtOH and MilliΩ water, the emission intensity increased substantially (***3-H***^**+**^ in Figure S5). We also observed a 45 nm hypsochromic shift, with λ_Em_ shifting from 565 nm in solution to 520 nm at the solid state (compare Figures 1, 3 with Figure S5). Both the increase in intensity and blue-shift of the fluorescence upon protonation are consistent with a disruption of π-π stacking interactions by augmented Coulombic repulsion and possible geometric rearrangement into a twisted conformation in the solid state. Such a configuration would also lead to less effective delocalization, which is likely responsible for the observed blue shift. It has also been suggested that blue-shifted emission at the solid state can occur as a result of lower reorganization energy in aggregates compared to solution (Wu et al., 2014). Moreover, the film can be dipped into a 40 mM ethanolic solution of NaOH for 30 min to deprotonate **3-H**^**+**^ and regenerate **3**, concomitantly lowering the fluorescence permanently, or until the system is once again subjected to an acid input (Figure 6). No fluorescence emission was observed for an unfunctionalized glass slide, before or after treatment with acid or base (Figure S6). Thus, the covalent attachment of **3** to a glass substrate permits long-term storage of the resulting emission readout (output) even after the removal of the system from acid or base inputs. This practical benefit generates a feedback loop by allowing the current state of the molecular switch to influence the next output state upon interaction with new inputs. As such, we can transduce the acidification of **3** into a “set” operation, with fluorescence at 520 nm defined as the output (O). Under this condition, the addition of IN1 (acid) can steadily “set” (*S* or *write*) the state of the molecular switch to *1*, and addition of IN2 (base) would “reset” (*R* or *erase*) the system to state *0*. Figure 6 illustrates the *S-R* mode transduced by the bistable solid state switch over several acid/base cycles, operating according to the INH convention set. Noticeably, the switching behavior at the solid state is representative of a single-output logic system, where only the fluorescence in the green channel can be modulated by the acid and base input combinations. In fact, no fluorescence is observed at higher wavelengths (Figure S5). Such behavior mirrors what is observed for the molecular switch **1** dissolved in a polar solvent (CH_3_CN), which is known to accelerate internal conversion and consequently quenches the fluorescence of the unprotonated species. It is possible that residual hydroxyl groups remaining on the glass surface after treatment with piranha solution are responsible for the relatively high polarity of the local environment, which commonly decreases the emission intensity, and hypsochromically shifts the absorption (compare Figures S1, S2, S4; Wagner et al., 2018). The utilization of a polar protic solvent (EtOH) for the immersion solutions would also have maintained a polar environment during the switching cycles. This rationale is also supported by the fact that a methoxy analog of compound **1** (Baruah et al., 2006) was noted to be almost completely quenched in MeOH.

## Conclusions

The growing demand for smaller and faster digital devices continues to stimulate the exploration of innovative materials for information processing. Molecules that respond to changes in their environment can simulate the behavior of the fundamental components of integrated circuits. Particularly, fluorescent chemosensors are able to switch from a non-emissive state to a fluorescent one, or change the color of emission in response to chemical inputs. The mechanisms responsible for fluorescence switching require coupling a selective chemical reaction to a photophysical process (emission). The observable changes in the emission of these probes are the result of the complex interplay between excited state dynamics and environmental parameters.

Herein, a bistable unimolecular system based upon the covalent conjugation between a boron-dipyrromethene chromophore and 4-(dimethylamino)benzaldehyde was synthesized in two steps and its environmentally responsive optical characteristics were investigated by steady-state absorption and emission spectroscopy. The system displays solvent and pH dependent emission in solution: the fluorescence quantum yield decreases in a polar aprotic solvent (CH_3_CN) and experiences a large bathochromic shift compared to the molecule dissolved in non-polar aprotic solvent (C_7_H_8_). In both solvents, large spectral shifts are observed in both absorption and emission spectra upon protonation of the tertiary amine. Interconversion between the protonated and neutral forms can be induced by subjecting the system to an acid or base input, and can be monitored by measuring the fluorescence of the two forms. Interplay between the two input stimuli can be exploited to transduce logic operations at the molecular level. Remarkably, the solvent dependency of the emission allows for the implementation of two different logic operators within the same system. Specifically, the logic function executed by this molecular switch in CH_3_CN is equivalent to that of a combinatorial single–output circuit incorporating an INHIBIT gate; in contrast, a combinatorial dual–output INHIBIT/IMPLY gate can be accessed by dissolving the system in C_7_H_8_. In both cases, positive logic conventions can be used to encode binary digits into the chemical inputs and optical outputs. Thus, the molecular system can be easily reconfigured between two modes (logic functions) simply by changing the operating medium. Surface immobilization of the molecular switch onto a solid support instilled sequential (programmable) character and memory, where the resulting output data can be stored for extended periods of time even after removal of the inputs, demonstrating the potential for integration into solid state technology. While the goal of this work was to study the viability of a strategy for information communication at the molecular level rather than to focus on practical applications, this system presents a number of interesting features, such as: (i) all outputs are encoded by optical signals (fluorescence); (ii) output reading by fluorescence spectroscopy does not affect the state of the logic gate; (iii) reconfigurability (currently limited to the solution phase) increases efficiency by reducing the number of logic devices required to form a multifunctional circuit, and (iv) operability (set/reset) at the solid state is the first step toward buildable data memory that can be achieved by integrating single “switchable” cells to form a complex circuit, where acid or base inputs write data along the array, and the output of an upstream gate provides the input for a downstream gate in a functional device.

## Data Availability Statement

The datasets generated for this study are included in the article/Supplementary Material.

## Author Contributions

SI conceived the project and wrote the manuscript. SI, LT, and GH designed the experiments. LT performed the experiments and analysis. ND, GH, and SI supervised the project. All authors contributed to project discussions.

Supplementary Material: Absorption spectra; FTIR spectra; DR spectrum of a functionalized glass slide; Emission spectra of an unfunctionalized control slide; Synthetic schemes.

## Conflict of Interest

The authors declare that the research was conducted in the absence of any commercial or financial relationships that could be construed as a potential conflict of interest.

## References

[B1] Andréasson J. Pischel U. (2015). Molecules with a sense of logic: a progress report. Chem. Soc. Rev. 44, 1053–1069. 10.1039/c4cs00342j 25520053

[B2] Baruah M. Qin W. Flors C. Hofkens J. Vallée R. A. L. Beljonne D.. (2006). Solvent and pH dependent fluorescent properties of a dimethylaminostyryl borondipyrromethene dye in solution. J. Phys. Chem. A 110, 5998–6009. 10.1021/jp054878u 16671668

[B3] Baytekin H. T. Akkaya E. U. (2000). A molecular NAND gate based on Watson-Crick base pairing. Org. Lett. 2, 1725–1727. 10.1021/ol005873c 10880211

[B4] Coskun A. Deniz E. Akkaya E. U. (2005). Effective PET and ICT switching of boradiazaindacene emission: a unimolecular, emission-mode, molecular half-subtractor with reconfigurable logic gates. Org. Lett. 7, 5187–5189. 10.1021/ol052020h 16268534

[B5] Costero A. M. Parra M. Gil S. Gaviña P. (2018). “BODIPY core as signaling unit in chemosensor design,” in BODIPY Dyes - A Privilege Molecular Scaffold with Tunable Properties, eds. Bañuelos-Prieto R. Sola Llano J. (London: IntechOpen).

[B6] Daly B. Ling J. de Silva A. P. (2014). Information gathering and processing with fluorescent molecules. Front. Chem. Sci. Eng. 8, 240–251. 10.1007/s11705-014-1432-z

[B7] de Silva A. P. (2012a). Molecular and Supramolecular Information Processing. ed Katz E. Weinheim: Wiley-VCH Verlag GmbH & Co. KGaA.

[B8] de Silva A. P. (2012b). Molecular Logic-based Computation. Cambridge: Royal Society of Chemistry.

[B9] de Silva A. P. Uchiyama S. (2007). Molecular logic and computing. Nat. Nanotechnol. 2:399. 10.1038/nnano.2007.188 18654323

[B10] de Silva P. A. McClenaghan N. D. (2002). Simultaneously multiply-configurable or superposed molecular logic systems composed of ICT (internal charge transfer) chromophores and fluorophores integrated with one- or two-ion receptors. Chem. A Eur. J. 8, 4935–4945. 10.1002/1521-3765(20021104)8:21<4935::AID-CHEM4935>3.0.CO;2-2 12397595

[B11] Deschamps J.-P. Valderrama E. Terés L. (2017). Digital Systems. (Cham: Springer International Publishing).

[B12] Erbas-Cakmak S. Kolemen S. Sedgwick A. C. Gunnlaugsson T. James T. D. Yoon J.. (2018). Molecular logic gates: the past, present and future. Chem. Soc. Rev. 47, 2228–2248. 10.1039/C7CS00491E 29493684

[B13] Feringa B. L. Browne W. R. (2011). Molecular Switches. Weinheim: Wiley-VCH Verlag GmbH & Co. KGaA.

[B14] Ghatak K. P. Mitra M. (2019). Nanomaterials, Volume 1: Electronic Properties. (Berlin: Walter de Gruyter GmbH).

[B15] Gibson J. (2013). Electronic Logic Circuits. London: Routledge.

[B16] Hwang E. (2016). Digital Logic and Microprocessor Design with Interfacing. Boston: Cengage Learning.

[B17] International Roadmap for Devices Systems Executive Summary (2018). Available online at: https://irds.ieee.org/editions/2018

[B18] Jeong Y. Yoon J. (2012). Recent progress on fluorescent chemosensors for metal ions. Inorganica Chim. Acta 381, 2–14. 10.1016/j.ica.2011.09.011

[B19] Jiang X. J. Ng D. K. P. (2014). Sequential logic operations with a molecular keypad lock with four inputs and dual fluorescence outputs. Angew. Chemie. Int. Ed. 53, 10481–10484. 10.1002/anie.201406002 25078949

[B20] Kaur P. Singh K. (2019). Recent advances in the application of BODIPY in bioimaging and chemosensing. J. Mater. Chem. C 7, 11361–11405. 10.1039/c9tc03719e

[B21] Kulyk B. Taboukhat S. Akdas-Kilig H. Fillaut J. L. Karpierz M. Sahraoui B. (2017). Tuning the nonlinear optical properties of BODIPYs by functionalization with dimethylaminostyryl substituents. Dye. Pigment. 137, 507–511. 10.1016/j.dyepig.2016.10.045

[B22] Leen V. Dehaen W. (2012). Fluorescent indicators based on BODIPY. Chem. Soc. Rev 41, 1130–1172. 10.1039/C1CS15132K 21796324

[B23] Lipiansky E. (2012). Electrical, Electronics, and Digital Hardware Essentials for Scientists and Engineers. Hoboken, NJ: John Wiley & Sons, Inc.

[B24] Margulies D. Melman G. Felder C. E. Arad-Yellin R. Shanzer A. (2004). Chemical input multiplicity facilitates arithmetical processing. J. Am. Chem. Soc. 126, 15400–15401. 10.1021/ja0453329 15563165

[B25] Mathew P. T. Fang F. (2018). Advances in molecular electronics: a brief review. Engineering 4, 760–771. 10.1016/j.eng.2018.11.001

[B26] Reinsel D. Gantz J. Rydning J. (2018). The digitization of the world from edge to core - International Data Corporation (IDC). Available online at: https://www.seagate.com/files/www-content/our-story/trends/files/idc-seagate-dataage-whitepaper.pdf

[B27] Rurack K. Kollmannsberger M. Daub J. (2001). Molecular switching in the near infrared (NIR) with a functionalized boron-dipyrromethene dye. Angew. Chemie Int. Ed. 40, 385–387. 10.1002/1521-3773(20010119)40:2<385::AID-ANIE385>3.0.CO;2-F 29712397

[B28] Sarid D. Schechtman B. H. (2007). A roadmap for optical data storage applications. Opt. Photonics News 18, 32–37. 10.1364/OPN.18.5.000032

[B29] Shi D. (2015). Nanomaterials and Devices. Oxford: Elsevier.

[B30] Shiraishi Y. Tokitoh Y. Hirai T. (2005). A fluorescent molecular logic gate with multiply-configurable dual outputs. Chem. Commun. 8, 5316–5318. 10.1039/b510800d 16244740

[B31] Stojanovic M. N. Stefanovic D. Rudchenko S. (2014). Exercises in molecular computing. Acc. Chem. Res. 47, 1845–1852. 10.1021/ar5000538 24873234 PMC4063495

[B32] Sun L. Diaz-Fernandez Y. A. Gschneidtner T. A. Westerlund F. Lara-Avila S. Moth-Poulsen K. (2014). Single-molecule electronics: from chemical design to functional devices. Chem. Soc. Rev. 43, 7378–7411. 10.1039/c4cs00143e 25099384

[B33] Swaminathan H. Balasurbamanian K. (2019). Design of “turn-ON and turn-OFF” fluorescence switching based photonic logic gates through multiple input-output models by MoS2 quantum dots. J. Colloid Interface Sci. 540, 258–264. 10.1016/j.jcis.2019.01.004 30660078

[B34] Szaciłowski K. (2012). Infochemistry. Chichester: John Wiley & Sons, Ltd.

[B35] Vollath D. (2013). Nanomaterials: An Introduction to Synthesis, Properties and Applications, 2nd Edn Weinheim: Wiley VCH.

[B36] Wagner B. D. Arnold A. E. Gallant S. T. Grinton C. R. Locke J. K. Mills N. D. (2018). The polarity sensitivity factor of some fluorescent probe molecules used for studying supramolecular systems and other heterogeneous environments. Can. J. Chem. 96, 629–635. 10.1139/cjc-2017-0727

[B37] Wu Q. Zhang T. Peng Q. Wang D. Shuai Z. (2014). Aggregation induced blue-shifted emission-the molecular picture from a QM/MM study. Phys. Chem. Chem. Phys. 16, 5545–5552. 10.1039/c3cp54910k 24509542

[B38] Yu Y. H. Descalzo A. B. Shen Z. Röhr H. Liu Q. Wang Y. W.. (2006). Mono- and di(dimethylamino)styryl-substituted borondipyrromethene and borondiindomethene dyes with intense near-infrared fluorescence. Chem. - An Asian J. 1, 176–187. 10.1002/asia.200600042 17441053

[B39] Zhang B. (2018). Physical Fundamentals of Nanomaterials. Oxford: Elsevier.

