# Supplementary material for: A Reconfigurable, Dual-Output INHIBIT and IMPLICATION Molecular Logic Gate
Source: Front Chem. 2020 Jun 5;8:470. doi: 10.3389/fchem.2020.00470 (PMC7290064; doi:10.3389/fchem.2020.00470)
Supplement: Supplementary file 1 [file Data_Sheet_1.pdf]

*Supplementary Material*

# A Reconfigurable, Dual-Output INHIBIT and IMPLICATION Molecular Logic Gate

**Lavinia A. Trifoi[1], Gregory K. Hodgson[1], Nicholas P. Dogantzis[1], Stefania Impellizzeri[1*]**

[1]Laboratory for Nanomaterials and Molecular Plasmonics, Department of Chemistry and Biology, Ryerson University, Toronto, ON, Canada

*** Correspondence:**
Stefania Impellizzeri
simpellizzeri@ryerson.ca

## Content

**Figure S1.** Absorption spectra of **1**, **1-H$^+$**, **1-B** and **1-H$^+$B** in $CH_3CN$……………………………………….S2

**Figure S2.** Absorption spectra of **1**, **1-H$^+$**, **1-B** and **1-H$^+$B** in $C_7H_8$……………………………………….S2

**Figure S3.** FTIR spectra of **2** and **3**……………………………………………………………...………S3

**Figure S4.** Diffuse reflectance spectrum of **3** immobilized on a glass slide…....................................S3

**Estimation of number of dye molecules on functionalized glass slide**...........................................S4

**Figure S5.** Emission spectra of **3** immobilized on a glass slide before and after acidification………...S5

**Figure S6.** Emission spectra of unfunctionalized glass slide before and after acidification or basification…………………………………………………………………………S5

**Synthetic Schemes**..……………………………………………………….………………..S6-S7

**References**...........................................................................................................................S8

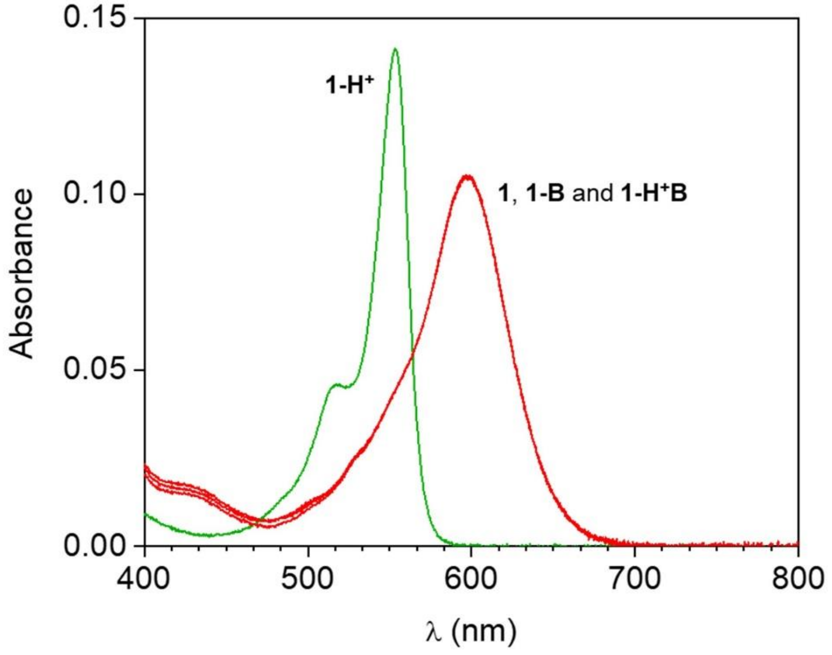


**Figure S1.** Absorption spectra of a $CH_3CN$ solution of **1** (1 μM, 20°C) before (**1**) and after the addition of 50 eq. $HClO_4$ (**1-H$^+$**), 50 eq. $NEt_3$ (**1-B**) and 50 eq. of $HClO_4$ + 50 eq. $NEt_3$ (**1-H$^+$B**).

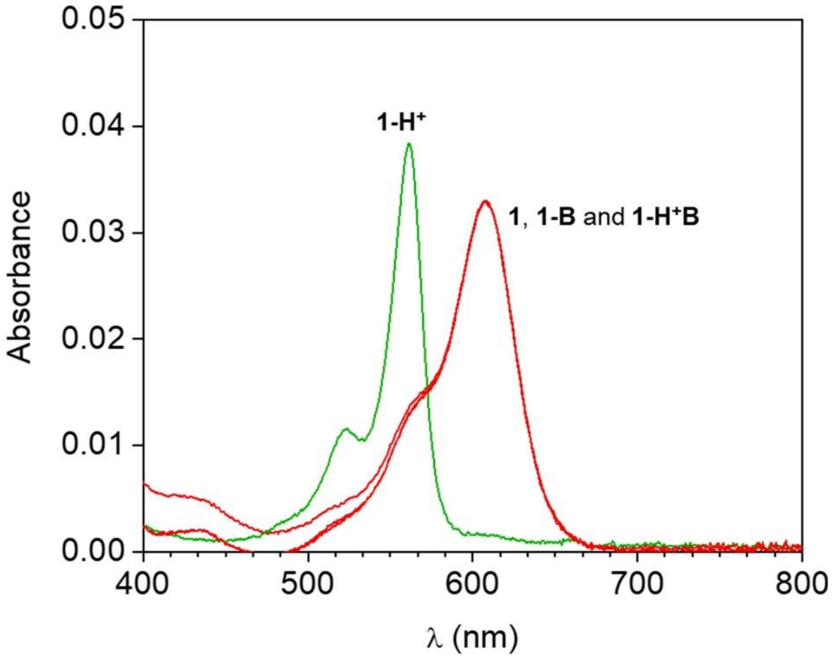


**Figure S2.** Absorption spectra of a $C_7H_8$ solution of **1** (1 μM, 20°C) before (**1**) and after the addition of 50 eq. $HClO_4$ (**1-H$^+$**), 50 eq. $NEt_3$ (**1-B**) and 50 eq. of $HClO_4$ + 50 eq. $NEt_3$ (**1-H$^+$B**).

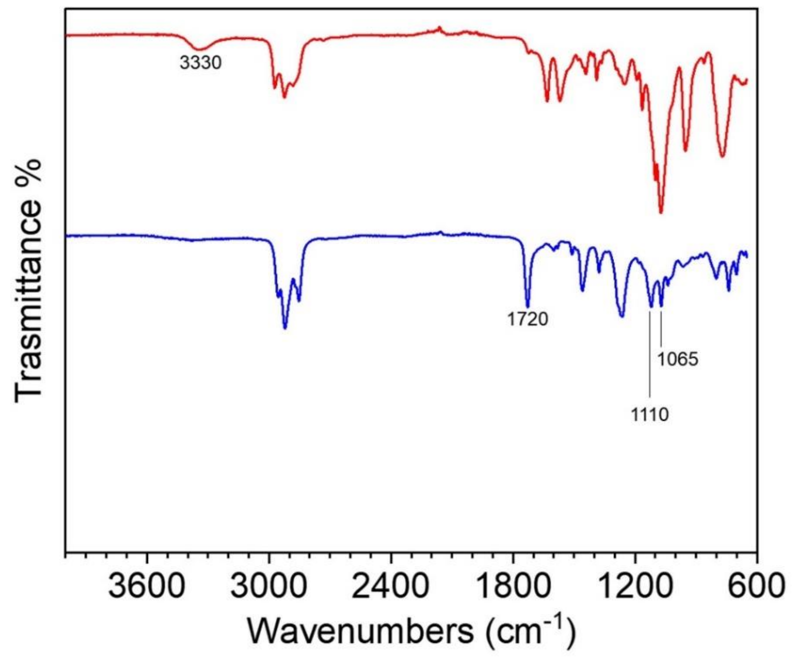


**Figure S3.** FTIR-ATR spectra of **2** (red trace, top) and **3** (blue trace, bottom).

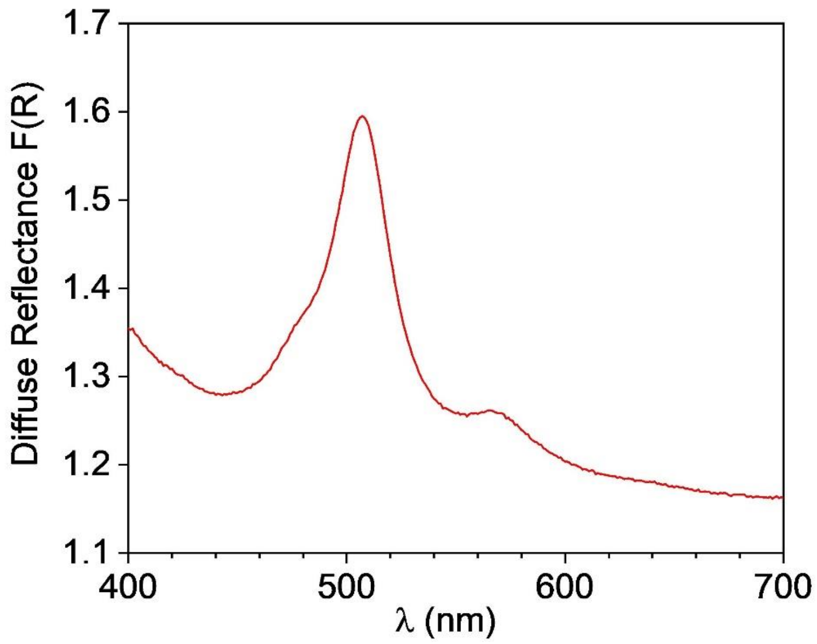


**Figure S4.** Diffuse reflectance spectrum of **3** immobilized onto a glass support (dried, 20°C).

**Estimation of number of molecules on functionalized glass slide**

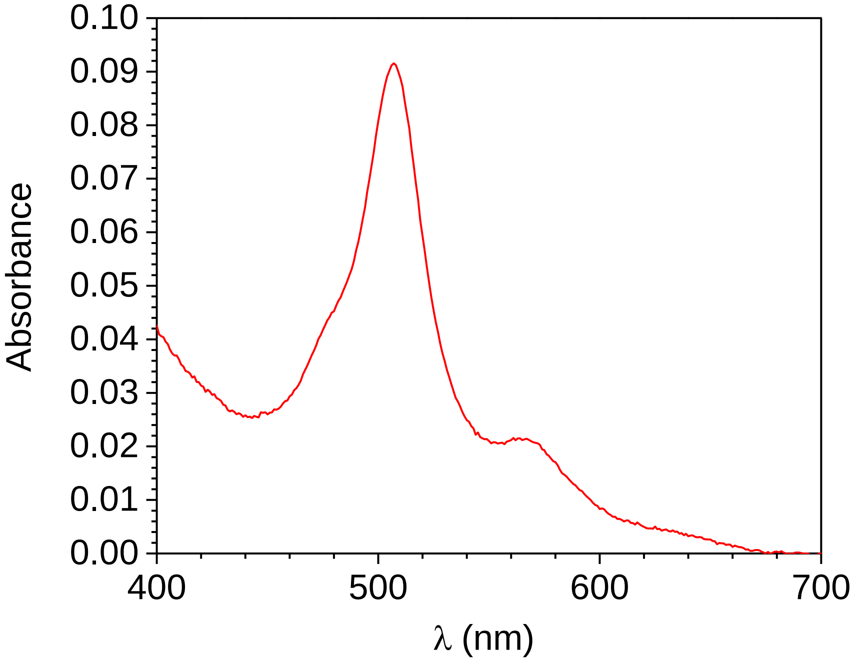


The approximate number of molecules of BODIPY present on a functionalized glass slide was estimated using the Beer-Lambert law along with the maximum absorbance of the sample (0.0916), which was obtained by collecting the same spectrum shown in Figure S4 in absorbance mode. Upon the basis of literature reports (Rurack et al., 2001) of the molar extinction coefficient of **1** in different environments (75,000 to 100,000 $M^{-1}$ $cm^{-1}$) as well as the dimensions of the glass slide, the number of molecules per unit area is estimated at between $8.6 \times 10^{13}$ and $1.1 \times 10^{14}$ molecules/$cm^2$. This estimate is on the same order of magnitude as literature reports of non-uniform monolayers in similar systems (Pan et al., 2006), and is also reasonable given that roughly $1.9 \times 10^{18}$ molecules/$cm^2$ were drop casted onto the glass slide prior to rinsing.

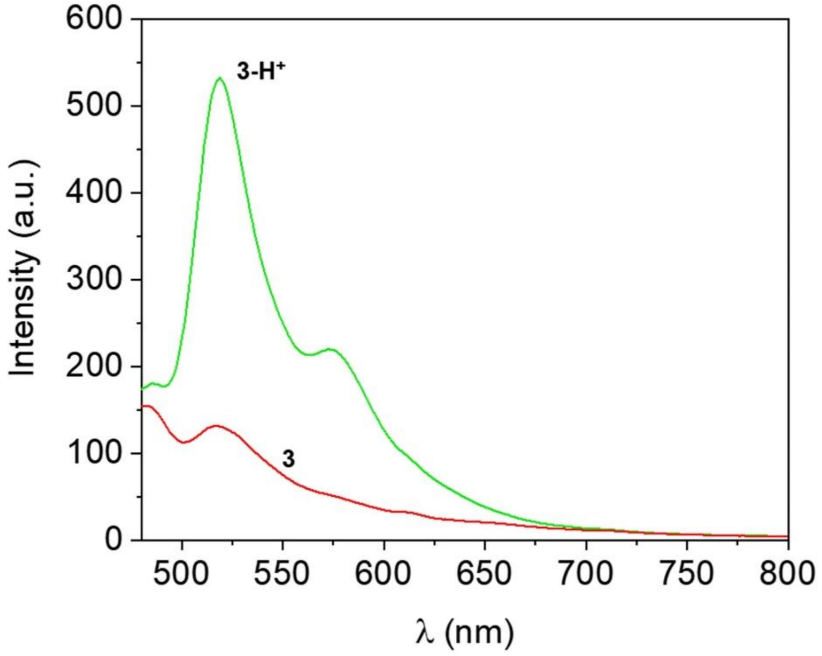


**Figure S5.** Emission spectra (20°C, $\lambda_{Ex}$ = 460 nm) of a glass slide functionalized with species **3** before (**3**) and after immersion into 0.015 M $HClO_4$ in EtOH (**3-H**$^+$).

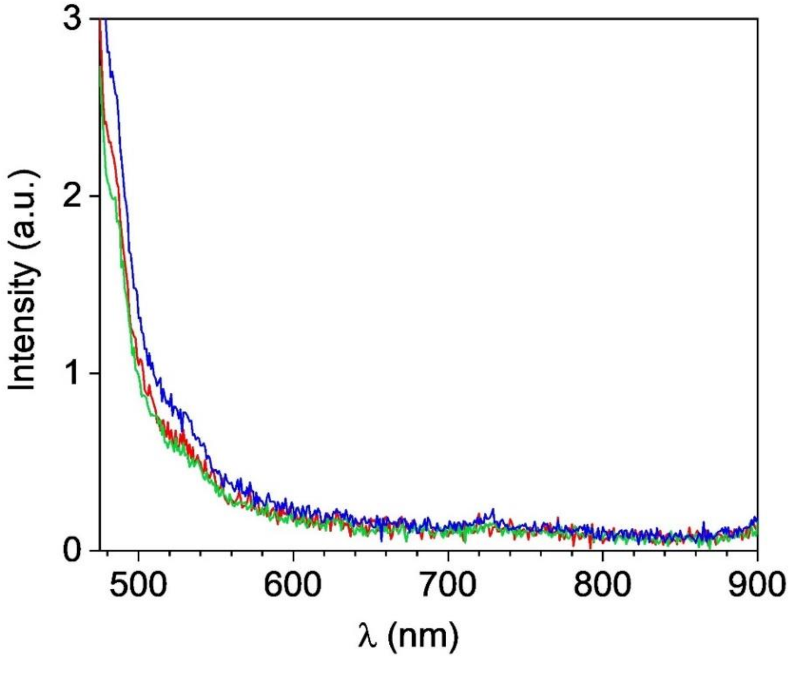


**Figure S6.** Control experiment showing the absence of fluorescence emission (20°C, $\lambda_{Ex}$ = 460 nm) for an unfunctionalized glass slide before (blue trace) and after (red and green traces) immersion into 0.015 M $HClO_4$ in EtOH (red trace) or 0.040 M NaOH in EtOH (green trace).

**Synthesis of 0.**

**0**

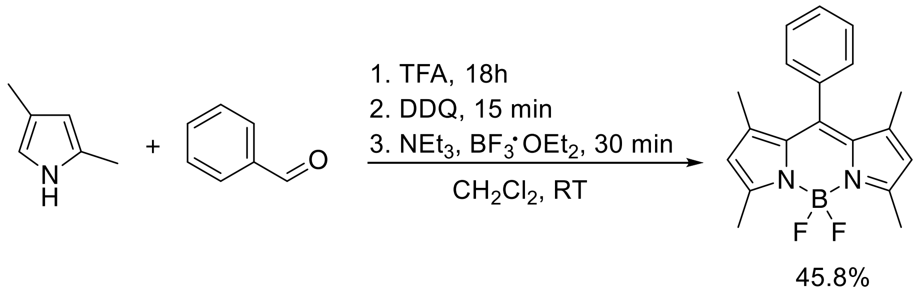


**Synthesis of 1.**

**1**

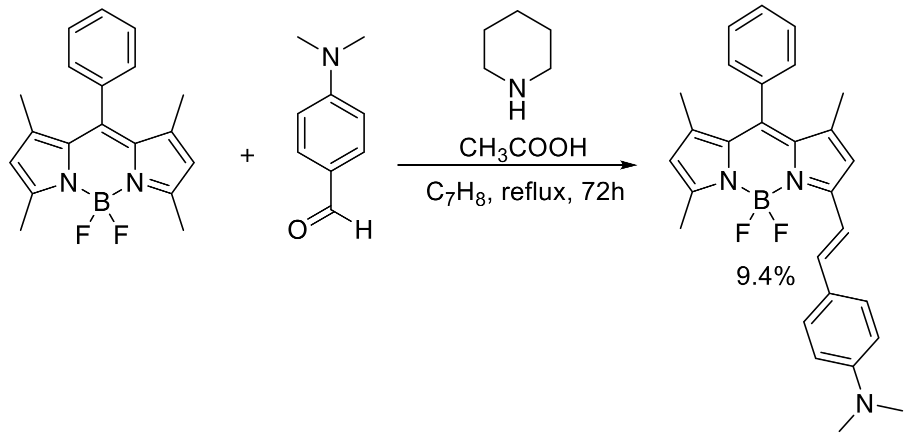


**Synthesis of 0b.**

**0b**

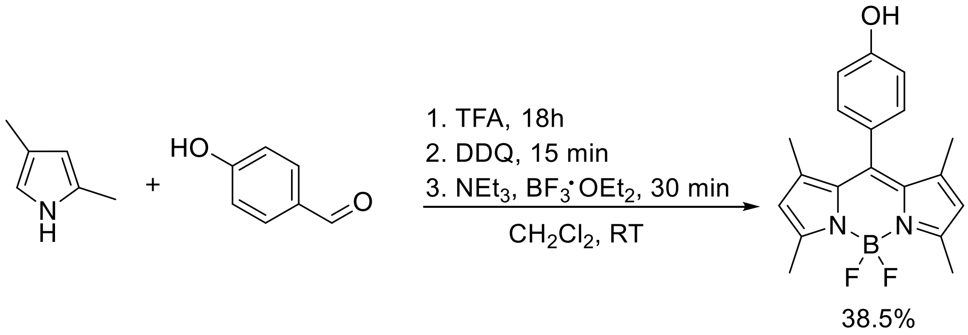

**Synthesis of 2.**

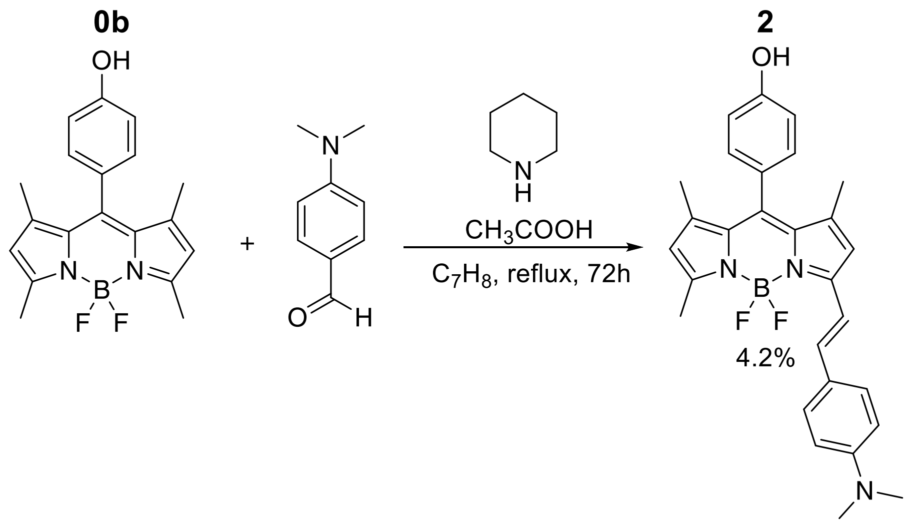


**Synthesis of 3.**

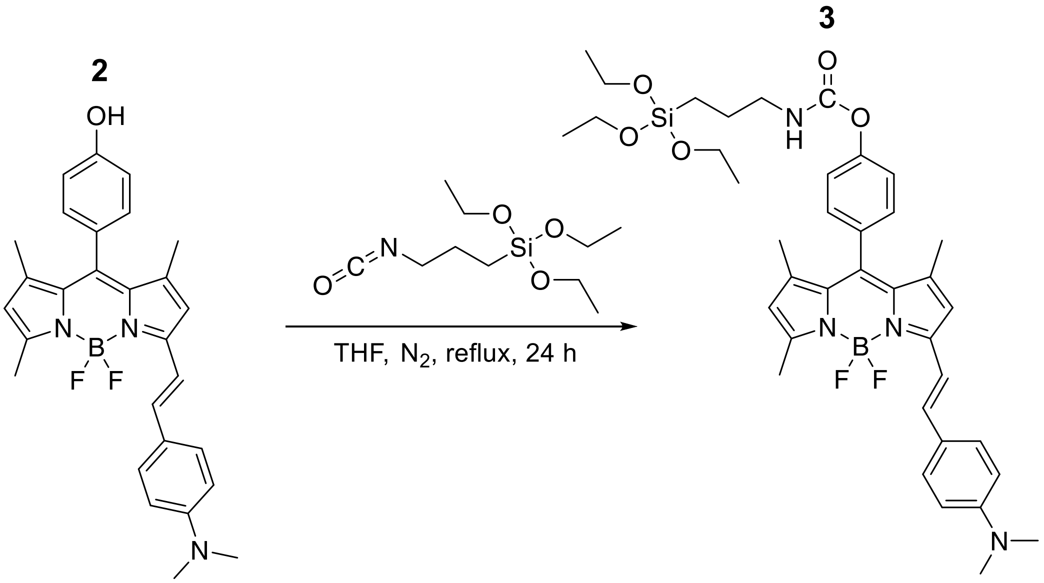

**References**

Pan, S., Wang, Z., and Rothberg, L. J. (2006). Enhancement of Adsorbed Dye Monolaeyer Fluoresecence by Silver Nanoparticle Overlayer. *J. Phys. Chem. B* 110, 17383–17387. doi:10.1021/jp063191m

Rurack, K., Kollmannsberger, M., and Daub, J. (2001). Molecular Switching in the Near Infrared (NIR) with a Functionalized Boron-Dipyrromethene Dye. *Angew. Chemie Int. Ed.* 40, 385–387. doi:10.1002/1521-3773(20010119)40:2<385::AID-ANIE385>3.0.CO;2-F
